# The relationship between social support and mental health problems during pregnancy: a systematic review and meta-analysis

**DOI:** 10.1186/s12978-021-01209-5

**Published:** 2021-07-28

**Authors:** Asres Bedaso, Jon Adams, Wenbo Peng, David Sibbritt

**Affiliations:** 1 College of Medicine and Health Sciences, School of Nursing, Hawassa University, Hawassa, Ethiopia; 2 Australian Centre for Public and Population Health Research, School of Public Health, Faculty of Health, University of Technology Sydney, Ultimo, NSW Australia

**Keywords:** Social support, Pregnancy, Mental illness, Anxiety, Depression, Self-harm, Systematic review, Meta-analysis

## Abstract

**Background:**

Pregnancy is a time of profound physical and emotional change as well as an increased risk of mental illness. While strengthening social support is a common recommendation to reduce such mental health risk, no systematic review or meta-analysis has yet examined the relationship between social support and mental problems during pregnancy.

**Methods:**

The PRISMA checklist was used as a guide to systematically review relevant peer-reviewed literature reporting primary data analyses. PubMed, Psych Info, MIDIRS, SCOPUS, and CINAHL database searches were conducted to retrieve research articles published between the years 2000 to 2019. The Newcastle–Ottawa Scale tool was used for quality appraisal and the meta-analysis was conducted using STATA. The Q and the I^2^ statistics were used to evaluate heterogeneity. A random-effects model was used to pool estimates. Publication bias was assessed using a funnel plot and Egger’s regression test and adjusted using trim and Fill analysis.

**Result:**

From the identified 3760 articles, 67 articles with 64,449 pregnant women were part of the current systematic review and meta-analysis. From the total 67 articles, 22 and 45 articles included in the narrative analysis and meta-analysis, respectively. From the total articles included in the narrative analysis, 20 articles reported a significant relationship between low social support and the risk of developing mental health problems (i.e. depression, anxiety, and self-harm) during pregnancy. After adjusting for publication bias, based on the results of the random-effect model, the pooled odds ratio (POR) of low social support was AOR: 1.18 (95% CI: 1.01, 1.41) for studies examining the relationship between low social support and antenatal depression and AOR: 1.97 (95% CI: 1.34, 2.92) for studies examining the relationship between low social support and antenatal anxiety.

**Conclusion:**

Low social support shows significant associations with the risk of depression, anxiety, and self-harm during pregnancy. Policy-makers and those working on maternity care should consider the development of targeted social support programs with a view to helping reduce mental health problems amongst pregnant women.

**Supplementary Information:**

The online version contains supplementary material available at 10.1186/s12978-021-01209-5.

## Plain language summary

Pregnancy is a significant event for reproductive-age women. It is supplemented by hormonal changes and can represent a time of increased risk for the occurrence of mental illness like depression, anxiety and self-harm. Providing good social support for the pregnant mother reduce this risk and prevent pregnancy complication and adverse birth outcome. However, no systematic review and/or meta-analysis has explored the associations between social support and mental illness (depression, anxiety, self-harm) among pregnant women. Therefore, this systematic review and meta-analysis aimed to examine the association between social support and mental illness (anxiety, depression, and self-harm) during pregnancy.

The review identified 67 relevant articles with 64,449 pregnant women, from PubMed, Psych Info, MIDIRS, SCOPUS, and CINAHL database. Of the total 67 articles, 22 articles included in the narrative review and 45 articles included in the meta-analysis. Among studies included in the narrative synthesis, a majority of them reported significant positive associations between low social support and antenatal depression, antenatal anxiety and self-harm during pregnancy. Further, the pooled estimates of the meta-analysis show that low social support had a significant positive association with antenatal depression (AOR: 1.18 (95% CI: 1.01, 1.41)) and antenatal anxiety (AOR: 1.97 (95% CI: 1.34, 2.92)). Therefore, maternal health professionals need to have discussions with pregnant women regarding the level and source of social support they receive. Maternal health professionals may also need to consider encouraging the social network of pregnant women to improve social support being given. Policy-makers and those working on maternity care should consider the development of targeted social support programs with a view to helping reduce mental health problems amongst pregnant women.

**Supplementary Information:**

The online version contains supplementary material available at 10.1186/s12978-021-01209-5.

## Background

Pregnant women are at increased risk of developing mental health problems such as depression, anxiety, and self-harm [1, 2]; a risk that can be exacerbated by different factors like financial and relationship issues and low social class [3–5]. The common mental health problems women experiencing during pregnancy are anxiety [6], depression [7] and self-harm [8]. Antenatal anxiety is defined as excess worries, concerns, and fears about pregnancy, childbirth, the health of the infant, and future parenting roles [9]. Individual studies have reported that the prevalence of antenatal anxiety range between 14 and 59% [10–13], while, a meta-analysis conducted on estimating the global prevalence of antenatal anxiety found that pooled prevalence of antenatal anxiety symptoms across all trimesters was 34.4% in low to middle-income countries and 19.4% in high-income countries [14].

Depression is the most prevalent mental health problem during pregnancy [15], characterized by symptoms such as depressed mood, low self-esteem, loss of interest, feelings of worthlessness, irritable mood, loss of appetite, feelings of fatigue, and poor concentration [16]. An umbrella review conducted on examining the global prevalence of antenatal depression reported 15–65%, and 17% pooled prevalence of antenatal depression in low to middle-income countries and high-income countries respectively based on ten identified systematic reviews [17].

Self-harm during pregnancy is one of the indirect causes of maternal death, especially among those who already developed mental health problems. For example in a study conducted in Bangladesh, among depressed pregnant women, nearly 14% admitted due to thoughts of self-harm [18] and in high-income countries, suicidal ideation is experienced by 3 to 33% of pregnant women [19, 20]. A global level review found that the prevalence of suicidal ideation during pregnancy and postpartum ranges from 5 to 14% [21].

Antenatal depression and anxiety negatively affect several obstetric and fetal outcomes and, if not effectively managed, can lead to pregnancy complications, postnatal mental health problems [22–27], and risk of impaired interaction between mother and infant [15, 28–30]. Mental illness during pregnancy is also associated with increased risk-taking behaviours such as smoking and the use of other substances that can thereby result in a poor quality of life of the mother [6, 31, 32].

One common strategy to help prevent or reduce pregnancy complications and adverse birth outcomes as a consequence of mental illness is to provide strong social support for the pregnant mother [6, 33, 34]. Social support is characterized by the degree to which social relationships fill specific needs (e.g. emotional, instrumental, affectionate, and/or tangible social support) or the degree of social integration [35, 36]. Social support is assumed to improve individuals' positive interactions that can help reduce depression, stress, and anxiety, and therefore reduce the risk of adverse pregnancy and birth outcomes [37]. Social support can also provide an additional suitable coping mechanism for pregnant women to handle stressful events [6, 37].

Different epidemiological studies have revealed that low social support is significantly associated with depression [38–40] anxiety [41, 42] and self-harm [43] during pregnancy. However, no systematic review and/or meta-analysis has been conducted to collate and critically review findings from individual studies; making the available evidence more accessible to decision-makers and providing an estimate of the magnitude of the associations between social support and mental health problems like depression, anxiety, and/or self-harm among pregnant women. In direct response to this significant research gap, this systematic review and meta-analysis aimed at examining whether low social support is associated with an increased risk of mental health problems during pregnancy. We hypothesized that low social support is significantly associated with depression, anxiety and/or self-harm during pregnancy.

## Methods

### Information source, search strategy and study selection process

This systematic review and meta-analysis was conducted and results were reported following the PRISMA (Preferred Reporting Items for Systematic Reviews and Meta-Analyses) checklist [44] (Additional file 1). This systematic review and meta-analysis protocol has been registered in PROSPERO (CRD42020155981). All peer-reviewed published articles were systematically searched through a number of electronic databases including PubMed, Maternal and Infant care database (MIDIRS), PsychINFO, SCOPUS, and CINAHL.

We used the following search terms and key words used for searching from the PubMed database: (((((((((("Depression" [Mesh] OR "Depressive Disorder" [Mesh] OR "Depressive Disorder, Major" [Mesh])) OR depression [Title/Abstract]) OR depressive symptom [Title/Abstract])) OR ((Anxiety disorder [Title/Abstract]) OR ((anxiety [Title/Abstract]) OR ("Anxiety" [Mesh] OR "Anxiety Disorders" [Mesh])))) OR ((((((((("Self-Injurious Behavior" [Mesh]) OR self-harm [Title/Abstract]) OR ("Self-Mutilation" [Mesh])) OR suicide [Title/Abstract]) OR "Suicide" [Mesh])) OR “Mental Health” [Mesh])) AND (((((("Social Support" [Mesh] OR "Psychosocial Support Systems" [Mesh])) OR social support [Title/Abstract]) OR Psychosocial support [Title/Abstract]) OR emotional support [Title/Abstract]) OR instrumental support [Title/Abstract])) AND ((((("Pregnancy" [Mesh]) OR "Pregnant Women" [Mesh])) OR pregnancy [Title/Abstract]) OR pregnant women [Title/Abstract]). For the other four electronic databases (CINAHL, MIDIRS, Psych INFO, and SCOPUS) specific database subject headings linked with the above terms and keywords were used. Search limits used in the databases include English literature and the period starting from January 1, 2000 to November 8, 2019. Also, we have manually searched the reference lists of included studies to identify additional articles. Using Covidence software [45], the identified publications were evaluated by their titles, abstract, duplication and full-text contents against the pre-specified inclusion and exclusion criteria.

We employed the PICO model to determine the eligibility for the study: population: (1) adults pregnant women aged ≥ 18 years*;* (2) intervention(s)/exposure(s) group: pregnant women who receive low social support*;* (3) comparison group(s): pregnant women who receive high/good social support*;* (4) outcomes: depression/depressive symptoms, anxiety disorder/anxiety symptoms and self-harm among pregnant women. The initial search and selection of studies were undertaken by AB. Full-text articles were later checked for their eligibility by two investigators (AB and WP). Disagreements were resolved through discussion with a third and fourth investigator (JA, DS) for the final selection of studies.

### Eligibility criteria

Studies that fulfil the following criteria were included. Firstly, studies that assessed and reported empirical data (primary or secondary) on the association between social support and depression, anxiety, or self-harm during pregnancy. Second, the types of study design are limited to observational studies such as cross-sectional, case–control, or cohort study design. Third, the participants of reported studies needed to be adult pregnant mothers whose age is 18 years old and above. Fourth, studies in which depression, anxiety and self-harm was confirmed by validated self-report screening instruments, structured interviews or other diagnostic criteria. The exclusion criteria’s were as follows: (1) Studies like clinical trials, literature reviews, commentaries, short communications, and letters to the editor, (2) studies that failed to report tool used to confirm the presence of mental health problems (depression, anxiety and self-harm) and the tool used to measure the social support given for pregnant women and (3) studies not published in the English language.

### Definition of outcome variables

In this study, mental health problems were operationalized as any diagnosed depressive disorders, general anxiety disorder, or suicidality (thoughts of self-harm, or suicidal attempt) according to standard diagnostic criteria such as the International Classification of Disease [46], the Diagnostic Statistical Manual of Mental Disorders (DSM) [47] or identified depressive symptoms or anxiety symptoms based on the valid screening tool.

### Definition of the exposure variable

In the current study, social support is broadly defined as the provision of emotional (e.g. caring), or informational (e.g. notifying someone of important information) support, instrumental (e.g. helping with housekeeping), tangible (e.g. practical support like financial aid), and/or psychological support for somebody by the social network of family members, friends, or community members [48].

### Quality appraisal and methods of data extraction

The modified version of the Newcastle–Ottawa Scale (NOS) was used to evaluate the methodologic quality (sample size, representativeness, comparability, non-response, ascertainment of outcome and statistical analysis) of the studies included in the current systematic review and meta-analysis [49]. Data extraction was independently completed from articles with good quality standards (NOS score ≥ 7 points) by two investigators (AB, WP) [50]. During the review process, any disagreement between the two investigators (AB, WP) was resolved through continuous discussion with review team members until consensus was reached. A specific form of data extraction format prepared in the Microsoft Excel spreadsheet (Additional file 2) was used. The following information was extracted from eligible full-text articles: author’s name, year of publication, country, sample size, study design, type of support, source of support, instrument employed, study setting, and measure of association and confidence interval.

### Data synthesis method

STATA IC version 16 statistical software was used to conduct a meta-analysis and estimate effect sizes. Studies were pooled to calculate pooled adjusted odds ratios and 95% CI using a random-effect model [51]. Adjusted odds ratios (AORs) were used as the preferred measure of association for meta-analysis, however, studies that analyse and report social support as a continuous exposure variable were reported in the narrative analysis. The narrative analysis was separately conducted for the association between social support and antenatal depression, antenatal anxiety, and antenatal self-harm. Among the studies included in the meta-analysis, most studies compared low social support with high/good social support. However, studies using low social support as a reference category were changed using the reciprocal method to maintain uniformity [52]. A meta-analysis of adjusted odds ratios for the association between low social support and outcome variables were calculated after log-transforming the estimates from eligible studies. If more than one outcome was reported from a single study each outcome was analysed independently.

### Publication bias, heterogeneity, and subgroup analysis

Possible publication bias was assessed through inspection of the funnel plot and Egger’s regression tests [53, 54]. The results of the tests suggested the existence of possible publication bias (p < 0.05 in Egger’s test), the final effect size (POR) was determined using Duval and Tweedie's Trim and Fill analysis in the Random-effects model [55]. The trim and fill analysis is a non-parametric method for approximating the number of missing studies that might exist and helps in reducing and correcting publication bias in meta-analysis. The presence of heterogeneity between studies was assessed using Q and the I^2^ statistics [51]. The I^2^ provides an estimate of the percentage of the variability in effect estimates that is due to heterogeneity rather than sampling error or chance differences. I^2^ statistics range from 0 to 100% and values of 25, 50 and 75% were considered to represent low, medium and high respectively [56]. A value of 0% indicates no observed heterogeneity while 100% indicates significant heterogeneity and a p-value < 0.05 was used to declare significant heterogeneity [56]. The possible sources of heterogeneity were identified using a univariate meta-regression model. Sub-group analyses were conducted based on study design, study setting, economic level of countries (low, middle and high income), median sample size and publication year. Sensitivity analysis was also undertaken to examine the effect of a single study on the overall effect size.

## Result

### Selection of studies

**Fig. 1:**
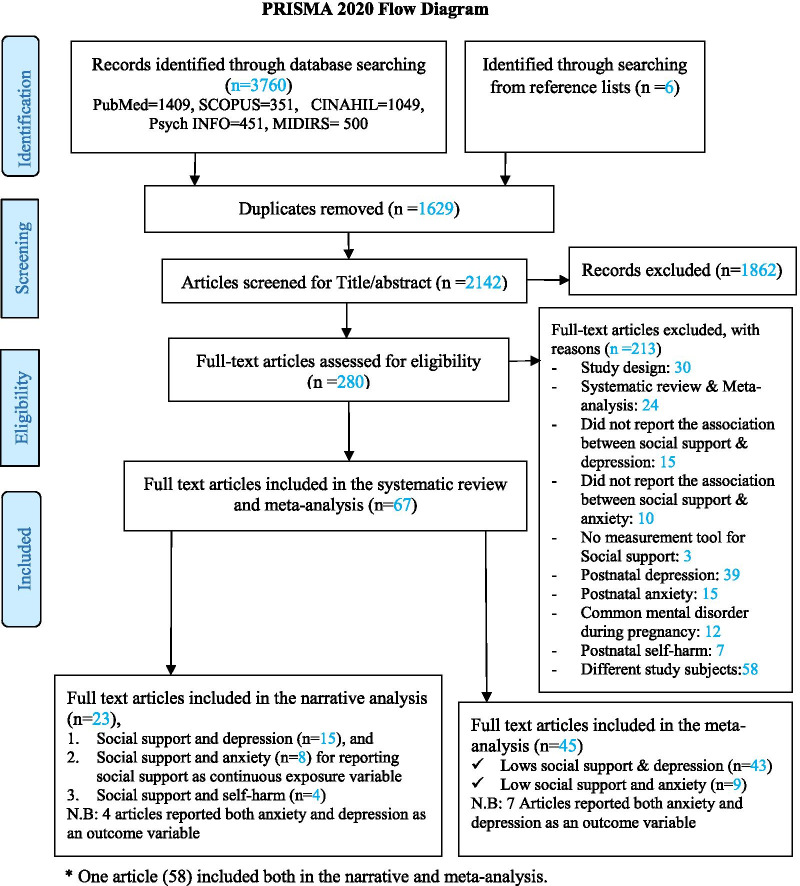
PRISMA flow chart of the study identification process for systematic reviews and meta-analyses, 2020

As indicated in Fig. 1, during the search strategy, 3760 papers were retrieved from five electronic databases. Also, an additional six citations were identified through a manual search of reference lists. After 1624 duplicates were removed, preliminary screening of the titles and abstracts of 2142 articles was conducted, and as a result a further 1862 articles were excluded. The remaining 280 articles met the criteria for full-text review with another 213 articles being excluded. Finally, 67 articles fulfilled the inclusion criteria and were included in the current systematic review and meta-analysis.

### Study characteristics

The characteristics of the included articles are presented in Table 1. Of the studies included in the present systematic review and meta-analysis, 21, 18, and 16 studies were conducted in high, middle, and low-income countries respectively representing 64,449 pregnant women. The majority of the studies, 31(47%), used the Edinburgh Postnatal Depression Scale (EPDS) for screening antenatal depression. The sample size of included studies ranges between 82 participants in the US [57] and 5337 participants in Canada [58].

**Table1:** Summary characteristics of studies investigating the association between social support and mental health problems (depression, anxiety and self-harm) during pregnancy (N = 66, from 2000–2019)

S. no.	Author, country, publication year	Type of support	Source of support	Sample size	Setting	Study design	Measurement		Measure of association
							Mental health problems	Social support	
1	Abujilban SK., et al., Jordan, 2013 [58]	General support	Family/non-family	218	Facility	Cross-sectional	Depression: EPDS	DUSOCS (12 item)	r = − 0.022, P > 0.05
2	Adewuya AO., et al., Nigeria, 2007 [59]	Emotional/instrumental support	Partner	181	Facility	Cross-sectional	Depression: EPDS	PSSS	AOR: 6.08 (1.42, 26.04)
3	Agostini F., et al., Italy, 2015 [60]	General support	Partner/family/friend	404	Facility	Longitudinal	Depression: EPDS	MSPSS	AOR: 1.06 (1.03, 1.08)
4	Akiki S., et al., UK, 2016 [61]	General support	Family	1992	Facility	Longitudinal	Anxiety: STAI	PSS	β = − 0.044, P = 0.029
			Partner						β = − 0.033, P = 0.0051
5	Anindyajati G., et al., Indonesia, 2017 [62]	General support	Partner/family	107	Community	Cross-sectional	Depression: LPGD	KD-24 item	AOR 0.21(0.05, 0.84)
6	Bayrampour H., et al., Canada, 2015 [63]	General support	Family/partner	3021	Facility	Longitudinal	Depression: EPDS	MOS-SSS (19 item)	AOR 3.09 (1.65, 5.78)
							Anxiety: STAI		AOR 3.37 (2.14, 5.33)
7	Belay YB., et al., Ethiopia, 2018 [64]	General support	Partner	363	Facility	Cross-sectional	Depression: BDI	MSSS	AOR 4.76 (1.51, 14.28)
8	Bernard O., et al., Jamaica, 2018 [65]	Emotional support	Partner	3571	Facility	Longitudinal	Depression: EPDS	MSPSS	AOR 3.14 (1.69, 5.84)
9	Biratu A., et al., Ethiopia, 2015 [66]	General support	Partner	422	Facility	Cross-sectional	Depression: EPDS	PSS	AOR 1.89 (1.06, 3.35)
10	Bisetegn TA., et al., Ethiopia, 2016 [67]	General support	Family/Partner	527	Community	Cross-sectional	Depression: EPDS	OSSS-3	AOR 1.57 (0.79,3.11)
11	Cankorur vs., et al., Turkey, 2015 [68]	Emotional/practical support	Family/partner	730	Facility	Longitudinal	Depression: EPDS	CPQ	AOR 1.07, (1.01, 1.15)
12	Chee C., et al., Singapore, 2005 [69]	General support	Family/partner	724	Facility	Longitudinal	Depression: EPDS	MOS-SSS (19 item)	AOR 2.53 (1.07–6.02)
13	Cheng E., et al., USA, 2016 [70]	General support	Friend/partner/relatives	1764	Facility	Longitudinal	Depression: EPDS	PSS	AOR: 3.1 (1.7, 5.7) (Project viva)
				877			Anxiety: STAI		AOR: 1.9 (1.1, 3.3) (Project access)
14	Clements AD., et al., USA, 2016 [71]	General support	Family/partner	106	Facility	Longitudinal	Depression: CESD	PPP	β = − 0.44, P < 0.001 (1^st^ trimester)
									β = − 0.33, P < 0.001 (2nd trimester)
15	Dibaba Y., et al., Ethiopia, 2013 [72]	General support	Family/partner	627	Community	Cross-sectional	Depression: EPDS	MSSS	AOR 4.34 (2.12, 9.1)
16	Dong X., et al., China, 2013 [73]	General support	Partner	520	Facility	Cross-sectional	Depression: EPDS	OSSS-3	AOR: 1.75 (0.16–19.28),
			Parents						AOR: 0.56 (0.06–5.13),
			Parents-in-law						AOR: 0.74 (0.24–2.23)
17	Dudas R., et al., Hungary, 2012 [74]	General support	Partner	1719	Community	Cross-sectional	Depression: LQ	MSPSS	AOR: 1.79(1.32–1.89)
18	Duko B., et al., Ethiopia, 2019 [75]	General support	Partner/parents/parents-in-law	317	Facility	Cross-sectional	Depression: EPDS	OSSS-3	AOR: 2.14 (1.49, 3.11)
19	Schetter DC., et al., Canada, 2016 [76]	General support	Family/partner	5271	Facility	Longitudinal	Anxiety: BIPS	MOS-SSS (19 item)	β = 0.08 (0.01, 0.15), P > 0.05
20	Fall A., et al., Canada, 2013 [57]	General support	Family	5337	Facility	Longitudinal	Depression: CES-D	ASSI	AOR: 4.47 (3.55–5.63)
21	Gao L., et al., China, 2019 [77]	Emotional/Instrumental support	Partner	278	Facility	Cross-sectional	Anxiety: SAS	PSSS	AOR: 2.86 (1.70, 4.83),
							Depression: EPDS		AOR: 2.56 (1.52, 4.30)
22	Golbasi Z., et al., Turkey, 2010 [78]	Emotional/Instrumental support	Family/partner/friend	258	Facility	Cross-sectional	Depression: EPDS	MSPSS	r = − 0.43; P < 0.001
23	Gourounti K., et al., Greece, 2013 [79]	General support	Family/friends/partner	165	Facility	Cross-sectional	Anxiety: STAI	SSQ-6	β = 0.131 (0.19, 2.37)
24	Hain S., et al., Germany, 2016 [80]	Emotional/instrumental support	Family/partner/non-family	297	Facility	Longitudinal	Depression: EPDS	F-SozU K-14	r = − 0.45, p < 0.01
25	Herbell K., et al., USA, 2019 [81]	General support	Family/partner	82	Facility	Cross-sectional	Depression: CESD	MOS-SSS (19 item)	β = − 0.751, P < 0.001
26	Heyningen T., et al., South Africa, 2017 [82]	Emotional/instrumental support	Friends	376	Facility	Cross-sectional	Anxiety: MINI	MSPSS	AOR: 1.05 (1.01, 1.09)
27	Jeong H., et al., South Africa, 2013 [83]	Emotional support	Mother	1262	Facility	Cross-sectional	Depression: EPDS	MSPSS	AOR: 1.5 (1.31–1.71)
28	Lau Y., et al., China, 2011 [84]	Emotional/tangible support	Family	1609	Facility	Cross-sectional	Depression: EPDS	ISEL	AOR: 1.9 (1.582, 2.520)
29	Lee AM., et al., Hong Kong, 2007 [85]	Emotional/instrumental support	Partner	357	Facility	Longitudinal	Anxiety: HADS	PSSS	AOR: 1.72 (1.05, 2.85)
							Depression: HADS		AOR: 1.69 (1.01, 2.85)
30	Li Y., et al., China, 2017 [86]	Emotional/instrumental support	Partner	240	Facility	Longitudinal	Depression: EPDS	PSSS	AOR 0.99 (0.94, 1.05)
31	Nath A.,et al., India, 2019 [87]	Emotional/instrumental support	Family/partner	380	Facility	Cross-sectional	Anxiety: PRT	MSPSS	AOR: 1.76 (1.04, 2.98)
32	Onah MN., et al., South Africa, 2016 [88]	Emotional/instrumental support	Family/friends/partner	376	Facility	Cross-sectional	Self-Harm: SIB	MSPSS	AOR: 1.07 (1.01, 1.15)
33	Pajulo M., et al., Finland, 2001 [89]	General support	Partner/parents/mother in-law/friend	391	Facility	Cross-sectional	Depression: EPDS	SSQ-12	AOR: 4.2 (0.9, 20.2)
34	Rashid A., et al., Malaysia, 2017 [90]	General support	Partner/parents/parents-in-law	3000	Facility	Cross-sectional	Depression: EPDS	OSSS-3	AOR: 2.16 (1.77, 2.64)
35	Rubertsson C.et al., Sweden, 2003 [91]	General support	Partner/parents/parents-in-law	608	Facility	Longitudinal	Depression: EPDS	OSSS-3	AOR: 6.9 (3.4, 13.9)
36	Shafaie FS., et al., Iran, 2017 [92]	General support	Family/Partner	372	Facility	Cross-sectional	Anxiety: DASS	PRQ- 85	r = − 0.456, p < .001
							Depression: DASS		r = − 0.642, p < .001
37	Sheeba B., et al., India, 2019 [93]	Emotional/instrumental support	Family/friends/partner	280	Facility	Longitudinal	Depression: EPDS	MSPSS	AOR: 1.785 (0.915, 3.48)
38	Sidebottom AC., et al., USA, 2017[94]	General support	Family/friend	2341	Facility	Longitudinal	Depression: PHQ-9	MSSI	AOR: 1.85 (1.31, 2.60)
39	Spoozak L., et al., USA, 2008 [95]	Emotional/instrumental support	Mother/partner	783	Facility	Cross-sectional	Depression: CIDI	MKSSI	AOR: 2.39 (1.63, 3.52)
40	Stewart RC., et al., Malawi, 2014 [96]	Emotional/instrumental support	Family/partner/friend	503	Facility	Cross-sectional	Depression: DSM IV	MSPSS	AOR 1.11 (1.04, 1.17)
41	Xian T., et al., China, 2019 [97]	General support	Family/partner	1220	Facility	Longitudinal	Anxiety: HAMA	SSRS-10	AOR: 5.09 (2.41, 10.77)
							Depression: SDS		AOR 3.18 (1.46, 6.96)
42	Verreault N., et al., Canada, 2014 [98]	General support	Family/partner/friends	364	Facility	Cross-sectional	Depression: EPDS	MOS-SSS (19 item)	β: − 0.32, P < 0.001
43	Woldetensay YK.,et al., Ethiopia, 2018 [99]	General support	Family/friends/partner	4680	Community	Longitudinal	Depression: PHQ-9	MSSS	AOR: 1.63 (1.31–2.02)
44	Yanikkerem E., et al., Turkey, 2013 [100]	Emotional/instrumental support	Partner	651	Facility	Cross-sectional	Depression: BDI	PSSS	β = 2.42, (0.707, 4.135)
45	Zeng Y., et al., China, 2015 [101]	Emotional/instrumental support	Family/partner/friend	292	Facility	Cross-sectional	Depression: SDS	SSRS-10	AOR 1.08 (1.03, 1.13)
46	Sahile MA., et al., Ethiopia, 2017 [102]	General support	Partner/parents/parents-in-law	231	Facility	Cross-sectional	Depression: BDI	OSSS-3	AOR: 2.63 (0.34, 20
47	Records C., et al., USA, 2007 [103]	Emotional/instrumental support	Family/friends/partner	139	Facility	Cross-sectional	Depression: CESD	MSPSS	β = 1.64, P < 0.001
48	Marchesi C., et al., Italy, 2014 [104]	General support	Family/friends/partner	277	Facility	Longitudinal	Anxiety: HADS	ASSI	AOR: 4.2 (1.1, 15.5)
49	Waqas A., et al., Pakistan, 2015 [105]	General support	Family/friend/ partner/others	500	Facility	Cross-sectional	Anxiety-HADS	SPS	r = − 0.433, P < 0.001
							Depression-HADS		r = − 0.453, P < 0.001
50	Westdahl C., et al., USA, 2007 [106]	General support	Family/parent	1047	Facility	Longitudinal	Depression: CESD	SSRS-10	AOR: 2.29 (1.21, 4.33)
51	Nasreen HE., et al., Bangladesh, 2011 [107]	General support	Partner/parents/parents-in-law	720	Community	Longitudinal	Depression: EPDS	OSSS-3	AOR: 2.23 (2.12, 3.62)
							Anxiety: STAI		β: -1.1447, P < 0.05
52	Leigh B., et al., Australia, 2008 [39]	General support	Family/friend/partner/others	367	Facility	Longitudinal	Depression: BDI	SPS	β = − 0.18, P < 0.001
53	Martini J., et al., Germany, 2015 [108]	General support	Family/friends/partner	306	Community	Longitudinal	Anxiety: CIDI-V	SSQ-12	AOR: 2.27 (1.42, 3.70)
							Depression: CIDI V		AOR: 2.43, (1.19, 5)
54	Rubertsson C. et al., Sweden, 2003 [91]	General support	Partner/parent/parents-in-law	3011	Facility	Longitudinal	Depression: EPDS	OSSS-3	AOR: 4.4 (2.7, 7.4)
55	Huang M., et al., Taiwan, 2019 [109]	General support	Family/friends/partner	158	Facility	Longitudinal	Anxiety: STAI	MSSS	β = − 0.79(− 1.16, − 0.42)
							Depression: EPDS		β = − 0.44 (− 0.63, − 0.24)
56	Jesse ED., et l, USA, 2005 [110]	General support	Partner/others	130	Facility	Cross-sectional	Depression: BDI	PPP	AOR:1(0.98,1.02),P > 0.05
57	Blaney NT., et al., USA, 2004 [111]	General support	Friend/partner/relatives	325	Facility	Cross-sectional	Depression: CESD	PSS	r = − 0.25, P < 0.001
58	Glazier RH., et al., Canada, 2004 [112]	Emotional/instrumental support	Family/friends/partner	2052	Facility	Longitudinal	Depression: CESD,	MSPSS	r = − 7.38, P < 0.01
						Longitudinal	Anxiety: STAI		r = − 7.34, P < 0.01
59	Senturk V., et al., 2011 [113]	General support	Partner	772	Facility	Cross-sectional	Depression: EPDS	CPQ	β = − 2.6 (− 3.6, − 1.7)
		Emotional support	Mother in-low						β = − 2.6,95%CI (-4.6,-1.9)
		Practical support							β = -0.8,95%CI (-1.4,-0.3)
60	Gausia k., et al., Bangladesh, 2009 [18]	General support	Mother in-low	361	Community	Cross-sectional	Depression: EPDS	PPP	AOR:2.41(1.31, 4.45)
			Partner						AOR: 8.26 (1.66, 41)
61	Shidhaye P., et al., India, 2017 [114]	General support	Friend/partner/relatives	302	Facility	Cross-sectional	Depression: EPDS	PSS	AOR: 3.33 (1.42, 5)
62	Hartley M., et al., South Africa, 2011 [115]	Emotional/practical support	Family/partner	1062	Facility	Longitudinal	Depression: EPDS	CPQ	AOR: 1.13 (1.03, 1.25)
63	Rwakarema M et al., Tanzania, 2017 [116]	General support	Family/friends/partner	397	Facility	Cross-sectional	Depression: EPDS	MSSS	AOR: 1.41 (0.60, 3.28)
64	Heyningen T et al., South Africa, 2016 [117]	Emotional/instrumental support	Family/friends/partner	376	Facility	Cross-sectional	Depression: EPDS	MSPSS	AOR: 1.14 (1.06, 1.22)
65	e Couto T., et al., Brazil, 2016 [43]	General support	Partner/parents/parents-in-law	255	Facility	Cross-sectional	Self-harm: MINI	OSSS-3	AOR: 1.75 (0.62, 5),
66	Pinheiro RT., et al., Brazil, 2012 [118]	Emotional/instrumental support	Family/friends/partner	871	Facility	Cross-sectional	Self-harm: MINI	MOS-SSS (7 item)	AOR: 3.03 (1.78, 5.26)
67	Supraja, TA., et al., 2016 India [119]	General support	Spouse, other family members, and friends	462	Facility	Cross-sectional	Self-harm: SBQ-R	MSSS-8 item	AOR: 1.17 (1.02, 2.35)

**Fig. 2:**
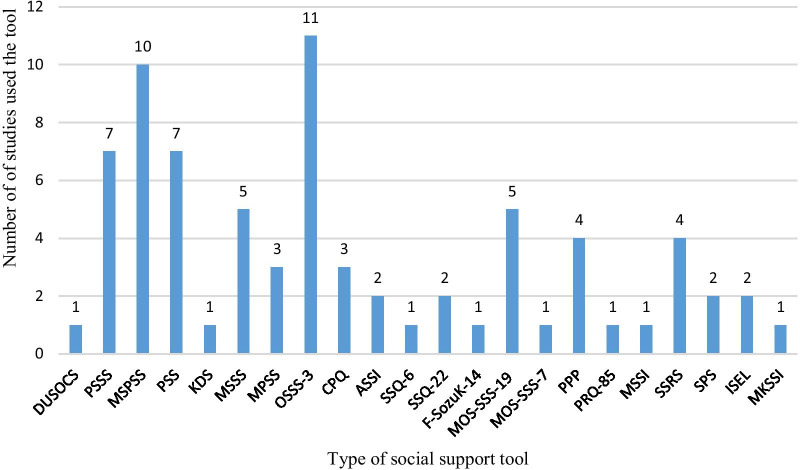
Types of social support tools used by the studies included in the current study

The articles included in the current systematic review and meta-analysis used 22 different valid measures of social support tools. From the total social support measures, the 3-item Oslo social support scale (OSSS-3) and Multidimensional Scale of Perceived Social Support (MSPSS) were the most dominant ones used by 11 and 10 studies respectively (Fig. 2). Details on the social support tools used and their reliability is outlined in Table 2.

**Table 2:** Social support tools used, concepts measured and their reliability

S. no.	Type of Social support tool used	Concepts measured	Cronbach Alpha
1	The Duke Social Support and Stress Scale (DUSOCS) (12 items) [119]	Family and non-family relationships in terms of the amount of social support they provide and number of supportive people	0.74
2	Multidimensional Scale of Perceived Social Support (MSPSS) (12 items) [120]	The subjective adequacy of emotional and instrumental social support from three different sources (family, friends, and partner)	0.83
3	Kuesioner Dukungan Sosial (KDS) (24 item) [121]	Support from husband, extended families from both sides, environmental support, mother's preparedness, and traditional rituals	0.788
4	Medical Outcomes Study Social Support Survey (MOS-SSS) (7 items) [35]	Perceived emotional and instrumental support from one’s social network	0.88
5	Medical Outcomes Study Social Support Survey (MOS-SSS) (19 items) [35]	Perceived emotional/informational support, tangible support, affectionate support, and positive social interaction	0.97
6	Oslo Social Support Scale(OSSS-3 item) [122]	Support received from husband, parents, and parents-in-law	0.88
7	Close Person Questionnaire (CPQ) [123]	Emotional support, practical support, and negative aspects of the relationship	0.85
8	Partner social support Scale (PSSS) [124]	Support received from a partner (emotional, instrumental and being dependent on partner)	0.89
9	Prenatal Psychosocial Profile (PPP) [125]	Social support from partner and others	0.71
10	Arizona Social Support Interview (ASSI) [126]	Availability of support from social network/family: instrumental, emotional, informative, normative, and companionship	0.7
11	Maternal Social Support Index (MSSI) (18 items) [127]	Support received from family, friend, and her feeling about the support. Also, the support received on routine home duties (watching children, doing other activities)	0.81
12	Modified Kendler Social support interview (MKSSI) (27 items) [128]	Emotional and instrumental support	0.68
13	Social Support rating scale (SSRS) (10 items) [129]	Objective support, subjective support, and support seeking behavior	0.76
14	Social provision Scale (SPS) (24 items) [130]	Intimacy, social integration, a reassurance of worth, and support from friends, family members, coworkers, community members, and so on	0.81
15	Social support Questionnaire (12 items) [131]	Emotional support, instrumental support, social integration and measure perceptions of social support and satisfaction with that social support	0.81
16	Social support Questionnaire-6 (SSQ-6 item) [132]	Availability of social support/number of supporters (SSQ-Network) and satisfaction with social support (SSQ-Satisfaction)	0.83
17	F-SozU K-14 (Fragebogen zur Sozialen Unterst€utzung; Social Support Questionnaire) (14 items) [133]	The perceived or anticipated emotional and instrumental support from one’s social environment	0.94
18	Interpersonal support Evaluation list (ISEL) (40 items) [134]	Perceived availability of aspects of social support like emotional, belonging, tangible, and self-esteem	0.74
19	Personal resource questionnaire (PRQ-85) (85 items) [135]	Assessment of the number of resources a person can count across life situations and a person's satisfaction with these resources	0.84
20	Perceived Social Support (PSS) (25 items) [136]	Social support from partner, friend, relatives, and co-worker	0.75
21	Social relationship Scale (SRS) [137]	Perceived availability of emotional and material support	0.87
22	Maternal social Support Scale (MSSS) (6 items) [138]	Level of perceived support from family, friends, and husband	0.74

Overall, from the total identified articles, 45 studies reported odds ratio (OR) as a measure of association between social support with antenatal depression and we included them in the meta-analysis presented here. Nine studies reported odds ratio (OR) as the measure of association between social support and antenatal anxiety and were included in the meta-analysis presented here. Twenty-three studies were included in the narrative analysis for analysing social support as a continuous variable [39, 59, 62, 72, 77, 79–82, 93, 99, 101, 106, 108, 110, 112–114]. Also, 4 studies [43, 89, 119, 120] that examined the relationship between self-harm and social support were included in the narrative analysis.

### Quality appraisal

From the included 67 articles, all scored greater or equal to 7 out of 10 on the NOS which are thereby considered as being good quality, which provides insights into the robustness of our meta-analysis (Additional file 3).

### Narrative analysis

#### Association between social support and antenatal depression

Fifteen studies that investigated the association [72, 82, 99, 101, 104, 110] or correlation [59, 79, 81, 93, 106, 112] between social support and antenatal depression were included in the narrative analysis. Among these 15 studies, 6 report a significant negative correlation between social support and antenatal depression [59, 79, 81, 93, 106, 112]. Also, a significant inverse relationship between social support and antenatal depression was reported by 8 studies [39, 72, 82, 99, 101, 104, 110, 114] and one study (conducted in Jordan) [59] reported no evidence of a significant correlation between social support and antenatal depression.

Among four studies conducted in the US, an inverse relation between social support and antenatal depression was reported from a longitudinal facility-based study conducted on 106 pregnant mothers, during the first trimester (β = − 0.44, P < 0.001) and second trimester (β = − 0.33, P < 0.001) [72]. Similarly, another facility-based cross-sectional study reported negative (β: − 0.751, P < 0.001) [82], and positive association (β = 1.64, P < 0.001) between social support and antenatal depression [104]. The fourth study which recruited pregnant women through a stratified random sampling technique revealed a negative correlation (n = 325, r = − 0.25, P < 0.001) between social support and antenatal depression [112].

Also, among three studies conducted in Turkey, negative moderate correlation between the EPDS score and perceived social support was reported from a facility-based cross-sectional study (n = 258, r = − 0.43; P < 0.001) [79]. Another facility-based cross-sectional study reported that social support was significantly related to depression (n = 655, β = 2.421, 95% CI (0.707, 4.135) [101]. In addition, another facility-based study indicated that support from husband (n = 772, β = − 2.6 (− 3.6, − 1.7), emotional support (β = − 2.6, 95% CI (− 4.6, − 1.9) and practical support (β = − 0.8, 95% CI (− 1.4, − 0.3)) from mother in low has inverse relation with antenatal depression [114].

Negative correlation between social support and antenatal depression was reported from longitudinal studies conducted in Germany (n = 297, r = − 0.45, p < 0.01) [81] and Canada (n = 2052, r = − 7.38, P < 0.01) [113]. Similarly, a negative correlation was reported from facility-based cross-sectional studies conducted in Iran (r = − 0.642, p < 0.001) [93] and Pakistan (r =  − 0.453, P < 0.001) [106]. A cross-sectional study conducted in Canada reported that social support was negatively associated with antenatal depression (n = 364, β: − 0.32, P < 0.001) [99].

A study conducted in Australia on consecutively selected pregnant mothers emphasized that good social support was negatively associated with depression during pregnancy (n = 367, β = − 0.18, P < 0.001) [39]. Similarly, a study from Taiwan reported an inverse relationship between social support and antenatal depression (n = 158, β =  − 0.44, 95% CI (− 0.63,  − 0.24), P < 0.05) [110]. Despite the above evidence of association, a cross-sectional study conducted in Jordan concluded that social support during pregnancy has no correlation with antenatal depression (r = − 0.022, P > 0.05) [59].

#### Association between social support and antenatal anxiety

Eight studies examined the association between social support and anxiety during pregnancy. Of which seven studies reported significant association [80, 108, 110] or correlation [62, 93, 106, 113] between social support and antenatal anxiety. However, one study conducted in Canada [77] reported no evidence of a significant association between social support and antenatal anxiety.

A longitudinal study conducted in the United Kingdom has shown that women receiving greater social support from their family reported feeling significantly less anxious; one standard deviation (SD) increase in social support (SS) from the family is associated with a 0.044 SD decrease in anxiety (P = 0.029). Also, a one SD increase in social support from the husband/partner was associated with a 0.033 SD decrease in STAI-State scores (P = 0.0051) [62].

Another finding from a longitudinal study in Bangladesh (n = 720) (β: − 1.144, P < 0.05) [108] and Taiwan (n = 158, (β =  − 0.79, 95% CI (− 1.16,  − 0.42), P < 0.05) [110] reported that social support during pregnancy was negatively associated with anxiety. A cross-sectional study conducted in Iran (n = 372) (r = − 0.456, p < 0.001) [93] and Pakistan (n = 500, r = − 0.433, P < 0.001) [106] among pregnant women revealed that there was a significant negative correlation between social support and anxiety during pregnancy. Similarly, a negative correlation was also reported from a longitudinal study conducted in Canada (n = 2052, r = − 7.34, P < 0.01) [113].

A facility-based study conducted in Greece on pregnant mothers concluded that there was no significant correlation between good social support and antenatal anxiety elation (n = 165, β = 0.131, 95% CI (0.19, 2.37), P > 0.05) [80]. A facility-based longitudinal study conducted in Canada reported social support did not have significant relation with antenatal anxiety (n = 5271, β = 0.08, 95% CI (0.01, 0.15), P > 0.05) [77].

#### Association between social support and self-harm during pregnancy

Due to the small number of studies examining self-harm and low social support among pregnant women, no meta-analysis was conducted on this specific association, thereby included in the narrative analysis. Three cross-sectional studies examined the association between social supports and self-harm during pregnancy. A cross-sectional study conducted in South Africa among randomly selected pregnant women reported a significant association between social support and suicidal ideation and behaviour (SIB) during pregnancy (n = 376, AOR: 1.07, 95% CI (1.01, 1.15), P < 0.05), suggesting a protective effect of good social support [89]. A cross-sectional study conducted in Brazil, which employed a consecutive sampling process to recruit pregnant women, reported that women with low social support were 3 times more likely to develop self-harm compared with their counterparts (n = 871, AOR: 3.03, 95% CI (1.78, 5.26) [119]. Finally, a study conducted in India among urban pregnant women, found that those who reported low perceived social support had a higher odds of developing current suicidal ideation (n = 462, AOR: 1.17, 95% CI (1.02, 2.35). However, a cross-sectional study, conducted in Brazil among 255 pregnant mothers, found no significant association between social support and self-harm (AOR: 1.75, 95% CI (0.62, 5) [43].

### Meta-analysis of the association between low social support and antenatal depression

Drawing upon data from 45 studies identified, a meta-analysis was conducted to examine the association between low social support and antenatal depression. From these 45 studies, 36 (80%) were conducted at the health facility level and 26 (57%) employed a cross-sectional study design. Also, 29 (64%) of the studies used the Edinburgh Postnatal Depression Scale (EPDS) as a screening tool to measure depression. From the identified 45 studies, a relatively large number of papers (20 [44.4%]) were published between the year 2016–2019 (Table 1).

**Fig. 3:**
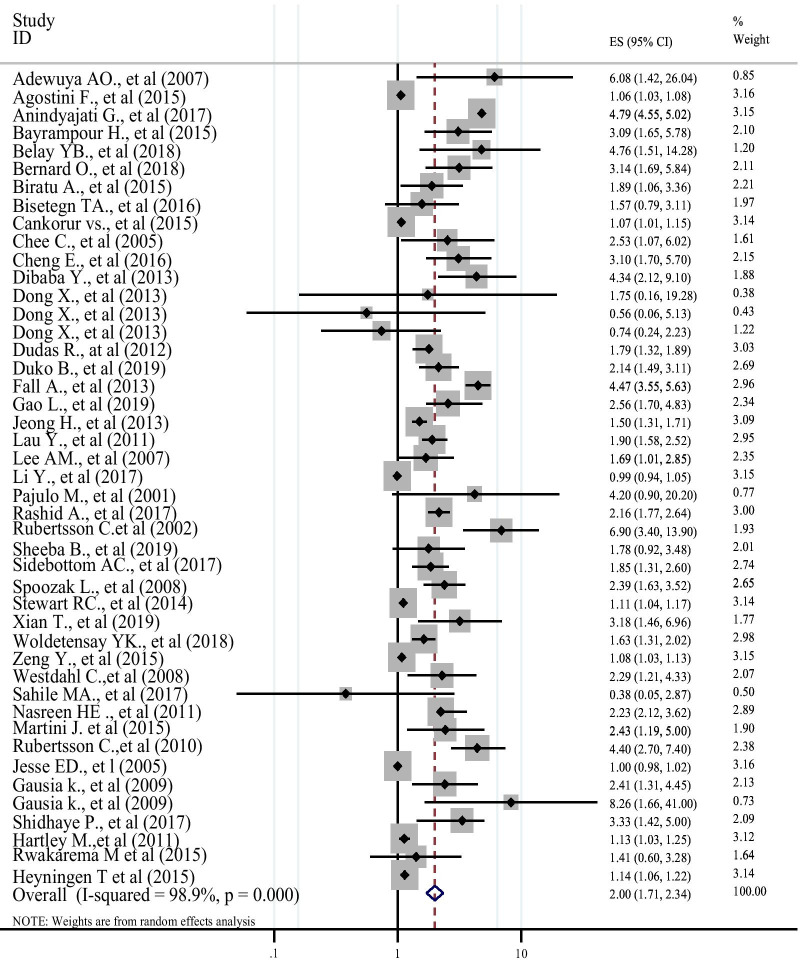
Forest plot indicating POR of low social support as a risk factor for antenatal depression

Except for eight studies [68, 74, 87, 90, 94, 103, 111, 117] all the remaining 37 studies included in the meta-analysis revealed low social support has a significant positive association with the risk of antenatal depression. The result of the meta-analysis showed low social support has a significant positive association with antenatal depression (AOR: 2.00 (95% CI: 1.71, 2.34) (Fig. 3). As the eggers test was found significant (p = 0.033), the final pooled effect size was corrected using Duval and Tweedie’s trim and fill analysis (AOR: 1.18 (95% CI: 1.01, 1.41). Due to the observed significant heterogeneity (I^2^ = 98.9%, Q = 3962.35, df = 44, P < 0.001) a random effect meta-analysis model was employed. To identify the possible sources of heterogeneity, variables such as publication year (Coefficient: − 0.019, P: 0.301) and sample size (Coefficient: − 0.0001, P: 0.019) were investigated via a univariate meta-regression model, and the sample size was statistically significant and identified as one of the possible sources of heterogeneity.

#### Subgroup analysis of the association between low social support and antenatal depression

Due to the reported high heterogeneity index among studies examining the association between low social support and antenatal depression, a subgroup analysis was conducted using characteristics like study setting, the income of countries, study design, sample size, publication year and tools used.

The subgroup analysis conducted based on the study setting revealed a higher POR of low social support among studies conducted at community setting (POR = 2.21, 95% CI: 1.25, 3.93, I^2^ = 99%, Q = 792.08, P < 0.001) compared with facility-based studies (POR = 2.21, 95% CI (1.25, 3.93), I^2=^93.0%, Q = 502.3, P < 0.001). In addition, a higher POR was estimated in the years 2000–2005, (POR = 4.37, 95% CI (2.20, 8.71, I^2^ = 36.0%, P < 0.001) followed by the years 2006–2010 (POR = 2.20, 95% CI (1.31, 3.71), I^2^ = 88.1%, P < 0.001). Regarding the median sample size, the POR of low social support was relatively higher among studies with a sample size greater than 520 (POR = 2.01, 95% CI (1.59, 2.55), I^2^ = 88.9%, P < 0.001) (Table 3).

**Table 3:** Subgroup analysis of odds ratios of low social support in the association between social support and antenatal depression (N = 45, 2000–2019), (random effect model)

Variable	No. of studies	Pooled AOR (95% CI)	Heterogeneity within the study (*I*^2^ and *Q*)	
			Q value	*I*^2^,* P-*value
*Study setting*				
Facility	36	1.63 (1.49, 1.77)	502.3	93.0%, p < 0.001
Community	9	2.21 (1.25, 3.93)	792.0	99.0%, p < 0.001
*Income of country*				
High-income	16	1.98 (1.72, 2.27)	367	95.9%, p < 0.001
Middle-income	14	1.33 (1.19, 1.50)	115.7	88.8%, p < 0.001
Low-income	15	2.26 (1.36, 3.76)	1431.5	99.0%, p < 0.001
*Study design*				
Cross-sectional study	26	1.84 (1.4, 2.41)	3534.2	99.3%, p < 0.001
Longitudinal study	19	2.1 (1.78, 2.48)	339.6	94.7%, p < 0.001
*Median sample size*^a^				
< 520	30	1.99 (1.63, 2.41)	3833.8	99.2%, p < 0.001
> = 520	15	2.02 (1.59, 2.55)	126.5	88.9%, p < 0.001
*Publication year*				
2000–2005	3	4.37 (2.20, 8.71)	3.13	36.0%, p = 0.209
2006–2010	7	2.20 (1.31, 3.71)	50.3	88.1%, p < 0.001
2011–2015	15	1.67 (1.43, 1.95)	295.8	95.3%, p < 0.001
2016–2019	20	2.06 (1.43, 2.97)	2598.4	99.3%, p < 0.001
*Depression assessment tool*				
Screening	41	2.07 (1.74, 2.46)	3940.9	99.0%, p < 0.001
Diagnostic	4	1.29 (1.09, 1.52)	19.25	84.4%, p < 0.001

^a^Cut of point is based on the median of sample size (median = 520)

#### Sensitivity analysis

A leave-one-out sensitivity analysis was conducted among studies examining the association between low social support and antenatal depression to help identify the effect of a single study on the overall pooled estimate. The sensitivity analysis using the random-effects model resulted in the POR ranges from 1.94 (95% CI: 1.66, 2.28) to 2.07 (95% CI: 1.71, 2.49). The sensitivity analysis shows that none of these studies was found to have substantially altered the overall results of the analysis.

### Meta-analysis of the association between low social support and antenatal anxiety

A meta-analysis was also conducted drawing upon data reported from 9 papers which examined the association between low social support and antenatal anxiety. From these studies, 8 were institution based cross-sectional studies and 6 (66.6%) used longitudinal study design and 5 reported data from high-income countries.

**Fig. 4:**
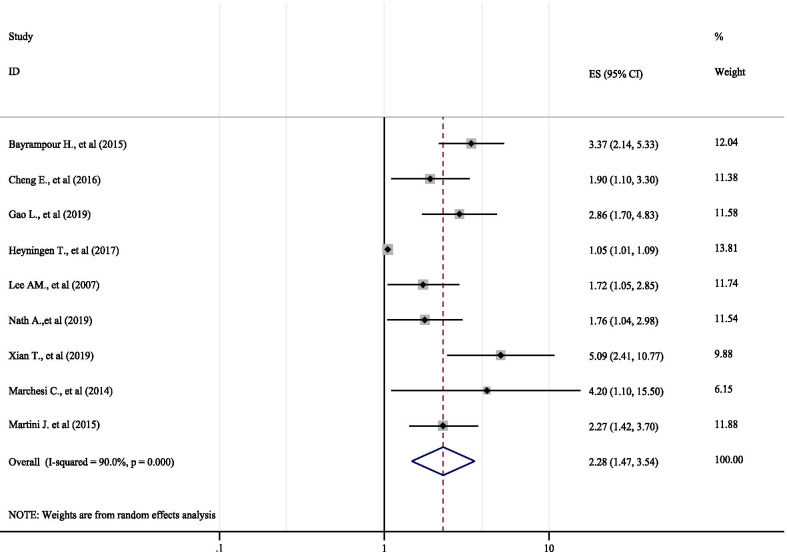
Forest plot indicating POR of low social support as a risk factor for antenatal anxiety

All the studies included in this meta-analysis found that low social support has a significant association with the risk of antenatal anxiety [64, 71, 78, 83, 86, 88, 98, 105, 109]. The pooled estimate found that low social support has a significant positive association with antenatal anxiety (AOR: 2.28 (95% CI: 1.47, 3.54) (Fig. 4). As the eggers test was found significant (p < 0.001), the final pooled effect size was corrected using Duval and Tweedie’s trim and fill analysis (AOR: 1.97 (95% CI: 1.34, 2.92). Since we found significant heterogeneity among the studies (I^2^ = 90.0%%, Q = 79.82, df = 8, P < 0.001) a random effect meta-analysis model was applied. To identify the possible sources of heterogeneity, variables such as publication year (Coefficient: 0.02, P: 0.688) and sample size (Coefficient: 0.0002, P: 0.261) were investigated using univariate meta-regression models, but none of these variables was found to be statistically significant.

#### Subgroup analysis of the association between low social support and antenatal anxiety

Due to the reported high heterogeneity index among studies examining the association between low social support and antenatal anxiety studies, a subgroup analysis was conducted using characteristics like study setting, the income of countries, study design, sample size, publication year and tools used.

The sub-group analysis undertaken based on the design of the study revealed a higher POR of low social support was among studies conducted using a longitudinal study design (POR: 2.59, 95% CI (1.87, 3.57), I^2^ = 44.2%, Q = 8.97, P = 0.11). In addition, a sub-group meta-analysis conducted based on the income of countries reported that higher POR of low social support was among high-income countries determined a POR of 2.34 (95% CI (1.76, 3.11), I^2^ = 23.6%, Q = 5.23, p = 0.264) (Table 4).

**Table 4:** Subgroup analysis of odds ratios of association between low social support and antenatal anxiety (N = 9, 2000–2019) (random effect model)

Variable	No. of studies	Pooled AOR (95% CI)	Heterogeneity within the study (*I*^2^ and *Q*)	
			Q value	*I*^2^,* P-*value
*Income of country*				
High-income	5	2.34 (1.76, 3.11)	5.23	23.6%, p = 0.264
Low and middle-income	4	2.15 (1.06, 4.38)	34.55	91.3%, p < 0.001
*Study design*				
Cross-sectional study	3	2.27 (1.46, 3.53)	17.68	88.7%, p < 0.001
Longitudinal study	6	2.59 (1.87, 3.57)	8.97	44.2%, P = 0.11
*Median sample size*^a^				
< 376	4	2.28 (1.72, 3.02)	2.77	0%, P = 0.429
> = 376	5	2.17 (1.16, 4.07)	49.53	91.9%, p < 0.001
Publication year				
2015+	7	2.27 (1.36, 3.78)	72.45	91.7%, P < 0.001
Before 2015	2	2.16 (1.007, 4.63)	1.53	34.7%, P = 0.216

^a^Cut of point is based on the Median of sample size (median = 376)

### Publication bias

**Fig. 5:**
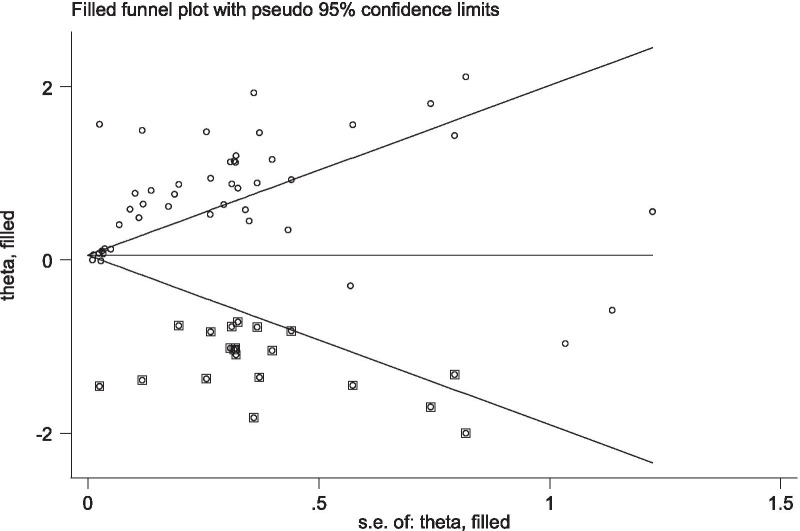
Tweedie’s and Duval’s trim and fill analysis on studies examining the association between low social support and antenatal depression

**Fig. 6:**
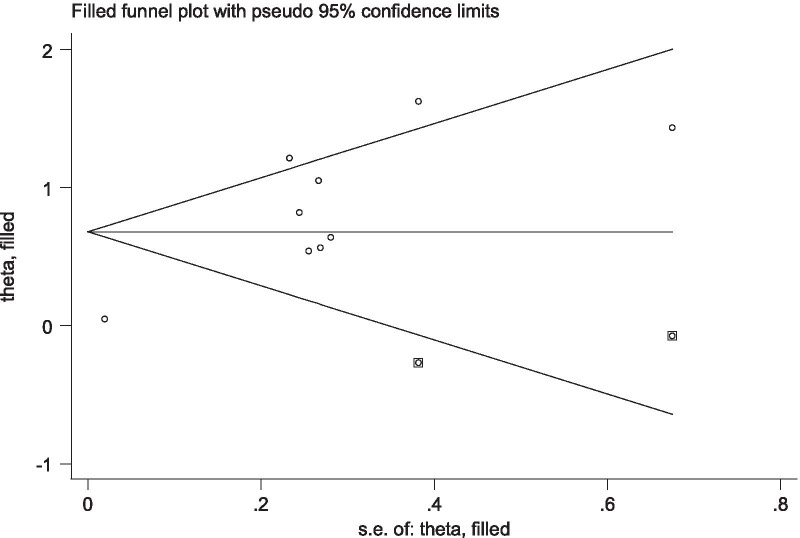
Tweedie’s and Duval’s trim and fill analysis on studies examining the association between low social support and antenatal anxiety

With regards to the literature reporting on the association of low social support with antenatal depression and antenatal anxiety, a funnel plot for both meta-analyses appeared asymmetrical indicating the presence of publication bias and Egger’s test for antenatal depression (P = 0.033) and antenatal anxiety (P < 0.001) also showed evidence of publication bias. In response, Duval and Tweedie’s trim and fill analysis was conducted. After adjusting for the publication bias, the trim and fill analysis reported an estimate of pooled AOR of low social support reduced from AOR: 2.00 (95% CI: 1.47, 3.54) to AOR: 1.18 (95% CI: 1.01, 1.41) for antenatal depression (Fig. 5) and from AOR: 2.28 (95% CI: 1.47, 3.54) to AOR: 1.97 (95% CI: 1.34, 2.92) for antenatal anxiety (Fig. 6).

## Discussion

Our study reports the findings of the first systematic review and meta-analysis examining the relationship between social support and mental illness (depression, anxiety, and self-harm) during pregnancy, revealing a number of interesting findings.

Our review identified that pregnant women who received low social support are more likely to develop mental illness compared to pregnant women who received good social support. Among studies included in the narrative synthesis, a majority of studies reported significant positive associations between low social support and antenatal depression [14/15 (93.3%) studies], low social support and antenatal anxiety [6/8 (75%) studies] and low social support and self-harm [3/4 (75%) studies] during pregnancy. Further, the pooled estimate of the meta-analysis shows that low social support had a significant positive association with antenatal depression and antenatal anxiety. Pregnant women with low social support may not have someone to confide in, obtain important information/advice from, or help reduce the negative emotions associated with a distressing situation, and as a result, they might be exposed to stress and may later develop depression [139]. Also, pregnant women with low social support are less satisfied with family and poor in interacting with the social environment, and as a result, they might be exposed to loneliness, become less in emotional and stress coping ability and later become more anxious [139, 140].

Support for our findings comes from various epidemiological studies conducted in general populations that have revealed low social support was associated with the onset and relapse of depression among individuals with a previous history of mental illness [141], and seasonal change of mood disorder in UK [142]. Also, low social support has a significant positive relationship with postnatal depression among a representative sample of Australian women [143].

A global level systematic review found that social support is also associated with improved mental health and decreased levels of depressive symptoms among female heads of households [144]. Good social support [145] may play a protective role against mental illness during pregnancy. Pregnant women who have good social support are more likely to have improved mental, psychological, and emotional health compared with their counterparts [6, 37]. Also, another research finding showed that individuals with constructive social relations and good social support enjoy more efficient communication skills, helping provide some protection from depression and other mental illnesses [146]. On the other hand, good social support protects people from illnesses [147] and can help provide an additional coping mechanism for stress [145].

A randomized control trial (RCT) examining the psychosocial benefits of a telephone support program for pregnant women in the metropolitan city on the South Island of New Zeeland revealed the intervention group at 34 weeks had lower stress scores, lower trait anxiety and less depressed mood than the control group [148]. However, another randomized controlled trial conducted in North East England involving low risk nulliparous pregnant women found that provision of additional telephone support by a midwife did not significantly reduce anxiety level (p = 0.68) [149]. Similarly, another RCT conducted in the US among pregnant women with a history of at least one spontaneous perinatal loss, found that providing intervention like home visits and support by nurses found no significant decrease in anxiety scores between the groups post-intervention (p = 0.66) [150].

As presented in sub-group analyses, among studies examining the association between low social support and antenatal depression, the pooled odds ratio of low social support was relatively higher among studies conducted in low-income countries compared with studies conducted in high-income countries. This might be due to the fact that most women living in low-income and middle-income countries face financial and economic challenges which might expose them to additional stress and social exclusion compared with pregnant women living in high-income countries [151]. Also, involvement in social activities may require money to attend events. So social exclusion and self-isolation of individuals from the social environment might lead them to feelings of loneliness and other psychological problems [152]. This concept was supported by a study conducted in Germany, which identified that socially disadvantaged persons more often report poor social networks and social support compared with their counterpart [153].

Finally, there was a significant level of heterogeneity amongst the studies examining the association between social support and either antenatal anxiety or antenatal depression. This high level of heterogeneity could be due to the different conceptualisation and measurement of social support employed in the studies. Our review identified, 22 different types of social support assessment tools used to measure social support. This shows a difference in the understanding of social support across many individuals and community members who were from different countries with different socio-economic settings. The lack of comprehensive agreement regarding the best method to measure social support is one of the identified challenge across the current literature [36]. As a result, work towards a unified social support measurement would be helpful.

### Implications for future research and clinical practice

Our review found that low social support has significant associations with the risk of mental health problems (depression, anxiety, and self-harm) during pregnancy. This suggests maternal health professionals need to have discussions with pregnant women regarding their level and source of social support. Policymakers and other relevant stakeholders should consider helping develop community-based social support programs for pregnant women to effectively integrate alongside other commonly used maternal health services. Reverse causation is possible between low social support and mental health problems during pregnancy. Therefore, to address the issue of reverse causation, future longitudinal studies, which can ensure the temporal order of events, is recommended. Finally, future interventional research is needed to further explore the effect of social support in preventing mental health problems during pregnancy.

### Limitations

There were some limitations to our study. The search was restricted to only include studies published in English language, which may lead to publication bias. Due to variations in diagnostic approaches, the assessments used for social support, depression and anxiety may be prone to measurement bias. However, we have addressed the issue of heterogeneity and publication bias during our analysis, which provides better estimates of the associations between social support and depression and anxiety during pregnancy.

## Conclusion

Low social support has significant associations with depression, anxiety, and self-harm during pregnancy. Strong social support may act to safeguard pregnant women from depression, anxiety, and self-harm. Maternal health professionals need to have discussions with pregnant women regarding the level and source of social support they receive and to also monitoring pregnant women’s mental health status if she is considered to have low social support. Maternal health professionals may also wish to consider encouraging the social network of pregnant women to improve social support being given. Policymakers and other relevant stakeholders should consider helping develop community-based social support programs for pregnant women that can be effectively integrated with other commonly used maternal health services. Finally, future research in this area should consider interventional studies that explore the effectiveness of social support in preventing mental illness during pregnancy.

## Supplementary Information

**Additional file 1.** PRISMA 2020 Checklist.

**Additional file 2.** Data extraction sheet used for studies examining the relationship between social support and mental health problems (depression, anxiety and self-harm) among adult pregnant mothers.

**Additional file 3.** Newcastle Ottawa (NOS) critical appraisal evaluation for Cross sectional and Cohort studies.

## Data Availability

All data generated or analyzed data during this review are included in this article and its additional file.

## Abbreviations

ASSI: Arizona social support interview
BAI: Beck anxiety inventory
BDI: Beck’s depression inventory tool
BIPS: Behaviour in pregnancy study tool
CESD: Centre for Epidemiologic Studies Depression Scale
CI: Confidence interval
CIDI: Composite international diagnostic intervention
CPQ: Close person questionnaire
DUSOCS: The Duke Social Support and Stress Scale
EPDS: Edinburgh Postnatal Depression Scale
HAMA: Hamilton Anxiety Scale (HAMA)
ISEL: Interpersonal support evaluation list
MINI: Mini International Neuropsychiatric Interview diagnostic interview
MKSSI: Modified Kendler Social Support Interview
MOSS: The Medical Outcomes Study Social Support Scale
MSOS-SSS: The 19-item Medical Outcomes Study Social Support Survey
MSPSS: Multidimensional Scale of Perceived Social Support
MSSI: Maternal Social Support Index
MSSS: Maternity Social Support Scale
NOS: Newcastle–Ottawa Scale
OSSS-3: 3 Items Oslo Social Support Scale
SBQ-R: Revised Suicidal Behaviour Questionnaire
