# Supplementary material for: The relationship between social support and mental health problems during pregnancy: a systematic review and meta-analysis
Source: Reprod Health. 2021 Jul 28;18:162. doi: 10.1186/s12978-021-01209-5 (PMC8320195; doi:10.1186/s12978-021-01209-5)
Supplement: Supplementary file 2 — Additional file 2. Data extraction sheet used for studies examining the relationship between social support and mental health problems (depression, anxiety and self-harm) among adult pregnant mothers. [file 12978_2021_1209_MOESM2_ESM.docx]

**Additional file 2:** Data extraction sheet used for studies examining the relationship between social support and mental health problems (depression, anxiety and self-harm) among adult pregnant mothers.

| **S.no** | **Author** | **Publication Year** | **Country** | **Sample size** | **Setting** | **Study design** | **Tool (Measures)** | | | | **Result**  **(Evidence of association)** | |
| --- | --- | --- | --- | --- | --- | --- | --- | --- | --- | --- | --- | --- |
|  |  |  |  |  |  |  | **Depression** | **Anxiety** | **Self-harm** | **Social support** | **AOR/r/β** | **CI (95%)** |
| 1 |  |  |  |  |  |  |  |  |  |  |  |  |
| 2 |  |  |  |  |  |  |  |  |  |  |  |  |
| 3 |  |  |  |  |  |  |  |  |  |  |  |  |
| 4 |  |  |  |  |  |  |  |  |  |  |  |  |
| 5 |  |  |  |  |  |  |  |  |  |  |  |  |
| 6 |  |  |  |  |  |  |  |  |  |  |  |  |
| 7 |  |  |  |  |  |  |  |  |  |  |  |  |
| 8 |  |  |  |  |  |  |  |  |  |  |  |  |
| 9 |  |  |  |  |  |  |  |  |  |  |  |  |
| 10 |  |  |  |  |  |  |  |  |  |  |  |  |
| 11 |  |  |  |  |  |  |  |  |  |  |  |  |
| 12 |  |  |  |  |  |  |  |  |  |  |  |  |
| 13 |  |  |  |  |  |  |  |  |  |  |  |  |
| 14 |  |  |  |  |  |  |  |  |  |  |  |  |
| 15 |  |  |  |  |  |  |  |  |  |  |  |  |
| 16 |  |  |  |  |  |  |  |  |  |  |  |  |
| 17 |  |  |  |  |  |  |  |  |  |  |  |  |
| 18 |  |  |  |  |  |  |  |  |  |  |  |  |
| 19 |  |  |  |  |  |  |  |  |  |  |  |  |
| 20 |  |  |  |  |  |  |  |  |  |  |  |  |
| 21 |  |  |  |  |  |  |  |  |  |  |  |  |
| 22 |  |  |  |  |  |  |  |  |  |  |  |  |
| 23 |  |  |  |  |  |  |  |  |  |  |  |  |
| 24 |  |  |  |  |  |  |  |  |  |  |  |  |
