# Supplementary material for: The relationship between social support and mental health problems during pregnancy: a systematic review and meta-analysis
Source: Reprod Health. 2021 Jul 28;18:162. doi: 10.1186/s12978-021-01209-5 (PMC8320195; doi:10.1186/s12978-021-01209-5)
Supplement: Supplementary file 3 — Additional file 3. Newcastle Ottawa (NOS) critical appraisal evaluation for Cross sectional and Cohort studies. [file 12978_2021_1209_MOESM3_ESM.docx]

**Additional file 3:** Newcastle Ottawa **(**NOS) critical appraisal evaluation for Cross sectional and Cohort studies

| **S.no** | **(Author, Year of pub.)** | **Representativeness of the sample** | **Sample size** | **Non respondent** | **Ascertainment of the exposure (risk factor)** | **Comparability (Confounding factors are controlled)** | **Assessment of outcome** | **Statistical Analysis** | **Total score** |
| --- | --- | --- | --- | --- | --- | --- | --- | --- | --- |
| 1 | Abujilban SK., et al, 2013 | 0 | 1 | 0 | 2 | 1 | 2 | 1 | 7 |
| 2 | Adewuya AO., et al, 2007 | 0 | 1 | 0 | 2 | 1 | 2 | 1 | 7 |
| 3 | Anindyajati G., et al, 2017 | 0 | 0 | 1 | 2 | 1 | 2 | 1 | 7 |
| 4 | Belay YB., et al, 2018 | 1 | 1 | 0 | 2 | 1 | 1 | 1 | 8 |
| 5 | Biratu A., et al, 2015 | 1 | 1 | 0 | 2 | 1 | 1 | 1 | 8 |
| 6 | Bisetegn TA., et al, 2016 | 1 | 0 | 0 | 2 | 1 | 2 | 1 | 7 |
| 7 | Dong X., et al, 2013 | 1 | 0 | 0 | 2 | 2 | 1 | 1 | 7 |
| 8 | Dudas R., at al, 2012 | 1 | 1 | 0 | 2 | 1 | 1 | 1 | 7 |
| 9 | Duko B., et al, 2019 | 1 | 0 | 0 | 2 | 1 | 2 | 1 | 7 |
| 10 | Gao L., et al, 2019 | 1 | 1 | 0 | 2 | 1 | 2 | 1 | 8 |
| 11 | Golbasi Z., et al, 2010 | 1 | 1 | 0 | 2 | 1 | 1 | 1 | 7 |
| 12 | Gourounti K., et al, 2013 | 1 | 1 | 1 | 2 | 1 | 1 | 1 | 8 |
| 13 | Herbell K., et al, 2019 | 1 | 1 | 0 | 2 | 1 | 1 | 1 | 7 |
| 14 | Jeong H., et al, 2013 | 1 | 1 | 0 | 2 | 1 | 2 | 1 | 8 |
| 15 | Lau Y., et al, 2011 | 1 | 0 | 0 | 2 | 1 | 2 | 1 | 7 |
| 16 | Pajulo M., et al, 2001 | 1 | 1 | 0 | 2 | 1 | 1 | 1 | 7 |
| 17 | Rashid A., et al, 2017 | 1 | 0 | 0 | 2 | 1 | 2 | 1 | 7 |
| 18 | Shafaie FS., et al, 2017 | 1 | 1 | 0 | 2 | 1 | 2 | 1 | 8 |
| 19 | Spoozak L., et al, 2008 | 1 | 0 | 0 | 2 | 1 | 2 | 1 | 7 |
| 20 | Stewart RC., et al, 2014 | 1 | 0 | 1 | 2 | 1 | 1 | 1 | 7 |
| 21 | Verreault N., et al, 2014 | 1 | 1 | 0 | 2 | 1 | 2 | 1 | 7 |
| 22 | Yanikkerem E., at al, 2013 | 1 | 1 | 0 | 2 | 1 | 2 | 1 | 8 |
| 23 | Zeng Y., et al, 2015 | 1 | 0 | 0 | 2 | 1 | 2 | 1 | 7 |
| 24 | Sahile MA., et al, 2017 | 1 | 1 | 0 | 2 | 1 | 2 | 1 | 8 |
| 25 | Records C., et al, 2007 | 1 | 1 | 0 | 2 | 1 | 2 | 1 | 8 |
| 26 | Waqas A., et al, 20115 | 1 | 0 | 1 | 2 | 1 | 2 | 1 | 8 |
| 27 | Jesse ED., et l, 2005 | 1 | 0 | 0 | 2 | 2 | 1 | 1 | 8 |
| 28 | Blaney NT., et al, 2004 | 1 | 0 | 0 | 2 | 1 | 2 | 1 | 7 |
| 29 | Gausia k., et al, 2009 | 1 | 1 | 0 | 2 | 1 | 2 | 1 | 8 |
| 30 | Shidhaye P., et al, 2017 | 1 | 1 | 0 | 2 | 1 | 2 | 1 | 8 |
| 31 | Hartley M., et al, 2011 | 0 | 1 | 0 | 2 | 1 | 2 | 1 | 7 |
| 32 | Rwakarema M et al, 2015 | 0 | 1 | 0 | 2 | 1 | 2 | 1 | 7 |
| 33 | Heyningen T et al, 2015 | 1 | 0 | 0 | 2 | 1 | 2 | 1 | 7 |
| 34 | Onah MN., et al, 2016 | 1 | 0 | 0 | 2 | 1 | 2 | 1 | 7 |
| 35 | Couto T., et al, 2015 | 0 | 1 | 0 | 2 | 1 | 2 | 1 | 7 |
| 36 | Pinheiro RT., et al, 2011 | 1 | 1 | 0 | 2 | 1 | 2 | 1 | 7 |
| 37 | Senturk V., et al, 2011 | 1 | 1 | 0 | 2 | 1 | 2 | 1 | 8 |
| 38 | Glazier RH., et al, 2004 | 1 | 0 | 0 | 2 | 1 | 2 | 1 | 7 |
| 39 | Dibaba Y., et al, 2013 | 0 | 1 | 1 | 2 | 1 | 1 | 1 | 7 |
| 40 | Heyningen T., et al, 2017 | 1 | 0 | 0 | 2 | 1 | 2 | 1 | 7 |
| 41 | Supraja, TA., et al , 2016 | 1 | 1 | 1 | 2 | 1 | 1 | 1 | 8 |

NOS critical appraisal evaluation for Cohort studies

| **S.no** | **(Author, year of pub.)** | **Selection domain** | | | | **Comparability domain** | | **Outcome domain** | | | **Total score* (9)** |
| --- | --- | --- | --- | --- | --- | --- | --- | --- | --- | --- | --- |
|  |  | Representativeness of exposed cohort (⋆) | Selection of non-exposed cohort (⋆) | Ascertainment of exposure (⋆) | Outcome of interest was not present at start of study (⋆) | Control for age or substance use or chronic medical illness (⋆) | Control for other variables (2nd important variables) (⋆) | Assessment of outcome (⋆) | Follow-up period (⋆) | Adequacy of follow up (⋆) |  |
| 1 | Agostini F., et al, 2015 | ⋆ | ⋆ | ⋆ | ⋆ | ⋆ | ⋆ | ⋆ | ⋆ | ⋆ | 9 |
| 2 | Akiki S., et al, 2016 | ⋆ | ⋆ | ⋆ | ⋆ | ⋆ | ⋆ | ⋆ | ⋆ | ⋆ | 9 |
| 3 | Bayrampour H., et al, 2015 | ⋆ | ⋆ | ⋆ | ⋆ | ⋆ | ⋆ | ⋆ | ⋆ | ⋆ | 9 |
| 4 | Bernard O., et al, 2018 | ⋆ | ⋆ | ⋆ | ⋆ | ⋆ | ⋆ | ⋆ | ⋆ | ⋆ | 8 |
| 5 | Cankorur vs., et al, 2015 | ⋆ | ⋆ | ⋆ | ⋆ | ⋆ | ⋆ | ⋆ | ⋆ | ⋆ | 9 |
| 6 | Chee C., et al, 2005 | ⋆ | ⋆ | ⋆ | ⋆ | ⋆ | ⋆ | ⋆ | ⋆ | 0 | 8 |
| 7 | Cheng E., et al, 2016 | ⋆ | ⋆ | ⋆ | ⋆ | ⋆ | ⋆ | ⋆ | ⋆ | ⋆ | 9 |
| 8 | Clements AD., et al, 2016 | ⋆ | ⋆ | ⋆ | ⋆ | ⋆ | ⋆ | ⋆ | ⋆ | ⋆ | 9 |
| 9 | Schetter DC., et al, 2016 | ⋆ | ⋆ | ⋆ | ⋆ | ⋆ | ⋆ | ⋆ | ⋆ | ⋆ | 8 |
| 10 | Fall A., et al, 2013 | ⋆ | ⋆ | ⋆ | ⋆ | ⋆ | ⋆ | ⋆ | ⋆ | ⋆ | 9 |
| 11 | Hain S., et al, 2016 | ⋆ | ⋆ | ⋆ | ⋆ | 0 | ⋆ | ⋆ | ⋆ | ⋆ | 8 |
| 12 | Lee AM., et al, 2007 | ⋆ | ⋆ | ⋆ | ⋆ | 0 | ⋆ | ⋆ | ⋆ | 0 | 7 |
| 13 | Li Y., et al, 2017 | ⋆ | ⋆ | ⋆ | ⋆ | ⋆ | ⋆ | ⋆ | ⋆ | 0 | 8 |
| 14 | Robertson C.et al, 2002 | ⋆ | ⋆ | ⋆ | ⋆ | ⋆ | ⋆ | ⋆ | ⋆ | ⋆ | 9 |
| 15 | Sheeba B., et al, 2019 | ⋆ | ⋆ | ⋆ | ⋆ | 0 | ⋆ | ⋆ | ⋆ | ⋆ | 8 |
| 16 | Sidebottom AC., et al, 2017 | ⋆ | ⋆ | ⋆ | ⋆ | ⋆ | ⋆ | ⋆ | ⋆ | ⋆ | 8 |
| 17 | Xian T., et al, 2019 | ⋆ | ⋆ | ⋆ | ⋆ | ⋆ | 0 | ⋆ | ⋆ | 0 | 7 |
| 18 | Woldetensay YK., et al, 2018 | ⋆ | ⋆ | ⋆ | ⋆ | ⋆ | ⋆ | ⋆ | ⋆ | ⋆ | 9 |
| 19 | Westdahl C., et al, 2008 | ⋆ | ⋆ | ⋆ | ⋆ | 0 | ⋆ | ⋆ | ⋆ | ⋆ | 8 |
| 20 | Nasreen HE ., et al, 2011 | ⋆ | ⋆ | ⋆ | 0 | ⋆ | ⋆ | ⋆ | ⋆ | 0 | 7 |
| 21 | Leigh B., et al, 2008 | ⋆ | ⋆ | ⋆ | ⋆ | ⋆ | ⋆ | ⋆ | ⋆ | ⋆ | 9 |
| 22 | Martini J. et al, 2015 | ⋆ | ⋆ | ⋆ | ⋆ | 0 | ⋆ | ⋆ | ⋆ | ⋆ | 8 |
| 23 | Rubertsson C.,et al, 2010 | ⋆ | ⋆ | ⋆ | ⋆ | ⋆ | ⋆ | ⋆ | ⋆ | 0 | 8 |
| 24 | Huang M., et al, 2019 | ⋆ | ⋆ | ⋆ | ⋆ | ⋆ | ⋆ | ⋆ | ⋆ | ⋆ | 9 |
| 25 | Marchesi C., et al, 2014 | ⋆ | ⋆ | ⋆ | ⋆ | ⋆ | ⋆ | ⋆ | ⋆ | 0 | 8 |
| 26 | Hartley M., et al, 2011 | ⋆ | ⋆ | ⋆ | ⋆ | ⋆ | ⋆ | ⋆ | ⋆ | 0 | 8 |

**NB: good quality score >7**
